# Respiratory Complex I dysfunction in cancer: from a maze of cellular adaptive responses to potential therapeutic strategies

**DOI:** 10.1111/febs.16218

**Published:** 2021-10-18

**Authors:** Manuela Sollazzo, Monica De Luise, Silvia Lemma, Licia Bressi, Maria Iorio, Stefano Miglietta, Sara Milioni, Ivana Kurelac, Luisa Iommarini, Giuseppe Gasparre, Anna Maria Porcelli

**Affiliations:** ^1^ Department of Pharmacy and Biotechnology (FABIT) Alma Mater Studiorum‐University of Bologna Bologna Italy; ^2^ Center for Applied Biomedical Research (CRBA) University of Bologna Bologna Italy; ^3^ Department of Medical and Surgical Sciences (DIMEC) Alma Mater Studiorum‐University of Bologna Bologna Italy; ^4^ Centro di Studio e Ricerca sulle Neoplasie (CSR) Ginecologiche Alma Mater Studiorum‐University of Bologna Bologna Italy; ^5^ Interdepartmental Center for Industrial Research (CIRI) Life Sciences and Technologies for Health Alma Mater Studiorum‐University of Bologna Ozzano dell'Emilia Italy

**Keywords:** adaptive responses, cancer metabolism, mitochondria, respiratory complex I, tumor microenvironment

## Abstract

Mitochondria act as key organelles in cellular bioenergetics and biosynthetic processes producing signals that regulate different molecular networks for proliferation and cell death. This ability is also preserved in pathologic contexts such as tumorigenesis, during which bioenergetic changes and metabolic reprogramming confer flexibility favoring cancer cell survival in a hostile microenvironment. Although different studies epitomize mitochondrial dysfunction as a protumorigenic hit, genetic ablation or pharmacological inhibition of respiratory complex I causing a severe impairment is associated with a low‐proliferative phenotype. In this scenario, it must be considered that despite the initial delay in growth, cancer cells may become able to resume proliferation exploiting molecular mechanisms to overcome growth arrest. Here, we highlight the current knowledge on molecular responses activated by complex I‐defective cancer cells to bypass physiological control systems and to re‐adapt their fitness during microenvironment changes. Such adaptive mechanisms could reveal possible novel molecular players in synthetic lethality with complex I impairment, thus providing new synergistic strategies for mitochondrial‐based anticancer therapy.

## Graphical abstract

Severe complex I (CI) dysfunction or its pharmacological inhibition have been shown to induce a quiescent phenotype in cancer cells. Despite this initial low‐proliferative status of tumor masses, these cancer cells may resume proliferation, activating adaptive molecular responses to overcome growth arrest. Such adaptive mechanisms could reveal possible novel molecular players in synthetic lethality with CI impairment, providing new findings for mitochondrial‐based anticancer therapeutic strategies.

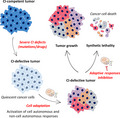

**Abbreviations**
αKG, α‐ketoglutarate; AIF: apoptosis‐inducing factor
AKT: protein kinase B
AML: acute myeloid leukemia
AMPK: AMP‐activated protein kinase
ATM: ataxia telangiectasia mutated
BNIP3: BCL2‐interacting protein 3
CI: complex I
ClpP: caseinolytic peptidase P
DNA‐PK: DNA‐dependent protein kinase
DRP‐1: dynamin‐related protein 1
EMT: epithelial–mesenchymal transition
ERRα: estrogen‐related receptor α
FAO: fatty acid β‐oxidation
FGF21: fibroblast growth factor 21
GDF‐15: growth/differentiation factor 15
GLUT: glucose transporter
GPX4: glutathione peroxidase 4
GSK3β: glycogen synthase kinase 3β
HIF‐1α: hypoxia‐inducible factor 1α
JNK1: c‐Jun N‐terminal protein kinase 1
MDP: mitochondrial‐derived peptide
MMP: metalloproteinase
mPTP: mitochondrial permeability transition pore
mTORC1: mammalian target of rapamycin 1
NF‐kB: nuclear factor kappa‐light‐chain‐enhancer of activated B cells
OCT: organic cation transporter
OXPHOS: oxidative phosphorylation
PGC‐1: peroxisome proliferator‐activated receptor gamma coactivator 1
PP2A: protein phosphatase 2
PRC: PGC‐1‐related coactivator
ROS: reactive oxygen species
SCO2: synthesis of cytochrome *c* oxidase 2
SPARC: secreted protein acidic and rich in cysteine
TAM: tumor‐associated macrophage
TCA: tricarboxylic acid
TIGAR: TP53‐induced glycolysis and apoptosis regulator
TME: tumor microenvironment

## Introduction

Mitochondria represent the hub for energy production, hosting the oxidative phosphorylation (OXPHOS) and the tricarboxylic acid (TCA) cycle, and actively contribute to macromolecule biosynthesis, reactive oxygen species (ROS) generation, and Ca^2+^ homeostasis, thus assuming a central position in signaling cascades and in the balance between cell survival and death [1]. Their organization as a dynamic network permits them to communicate with other intracellular organelles, such as the nucleus and the endoplasmic reticulum, and to act as stress sensors, thus modulating cell adaptation to microenvironmental changes during both physiological and pathological conditions. The primary function of mitochondria is to produce energy through the OXPHOS, whose impairment may arise from both nuclear (nDNA) and mitochondrial DNA (mtDNA) mutations, or epigenetic changes. Such OXPHOS defects have been widely involved in several human disorders, including cancer [2, 3, 4]. In particular, respiratory complex I (CI) dysfunction plays a controversial role during cancer onset and progression [5, 6]. Indeed, depending on the type and severity of mtDNA alterations, the impact of CI dysfunction on cancer cell growth and survival can be even opposite, leading to the definition of CI as an ‘*oncojanus’* [5, 6, 7, 8]. The neologism *oncojanus* was coined by our group in 2011 to describe *MT‐ND1* as a gene, encoding the CI ND1 subunit, that is able to differently impact on tumor progression depending on both its mutation type and load, a concept that was subsequently reinforced by correlating *MT‐ND1* mutations with diverse degrees of CI dysfunction [5, 6]. Such definition is now applied also to other non‐canonical oncogenes and tumor suppressor genes for which a double‐edged role in tumor initiation, progression, or response to therapy has been brought to light [9, 10, 11, 12, 13, 14]. With respect to tumor progression, mild CI defects, generally determined by missense and heteroplasmic mtDNA mutations, exhibit protumorigenic or neutral effects [7, 8, 9, 10]. On the other hand, severe CI dysfunction invariably hampers tumor progression and is accompanied by profound alterations of metabolic equilibrium, Ca^2+^ homeostasis, and intramitochondrial oxygen availability [5, 11, 15]. In cancer patients, such severe functional defects are generally caused by disruptive mtDNA mutations which are rare events often subjected to counterselection [16, 17, 18]. However, they represent the sole molecular hallmark of oncocytomas, a mostly indolent subtype of epithelial tumors characterized by a compensatory accumulation of deranged mitochondria (oncocytic or oxyphilic phenotype) [19]. In oncocytomas, the accumulation of homoplasmic disruptive mutations hampers tumor growth *in vivo* due to alterations of energy metabolism and hypoxic adaptation failure [20]. This mechanism has been generalized modeling different mtDNA and nDNA mutations in CI structural genes in different cancer models both *in vitro* and *in vivo* [5, 6, 11, 21]. During tumor progression, severe CI dysfunction initially results in a drawback for cancer cells keeping them in a low‐proliferative state similar to that of benign oncocytomas, thus identifying CI as a potential druggable target for new metabolic anticancer therapeutic strategies. Despite this, over time, it can be envisioned that some CI‐defective cancer cells may resume proliferation by triggering adaptive molecular feedbacks and atypical microenvironment responses to support survival [11]. These findings led us to hypothesize that the initial quiescent state may select those cells better capable to adapt to the metabolic alterations caused by CI dysfunction and to the tumor microenvironment (TME) selective pressures, thus reconstituting their proliferative abilities and progression toward malignancy.

In this review, we retrace the potential cell‐ and non‐cell‐autonomous adaptive responses that are activated in CI‐defective cancer cells. The dissection of such pathways may allow the identification of molecular players in synthetic lethality with impaired CI to be targeted synergistically to prevent the proliferation rescue of CI‐defective tumor cells.

## Cell‐autonomous responses in CI‐defective cancer cells

**Fig. 1:**
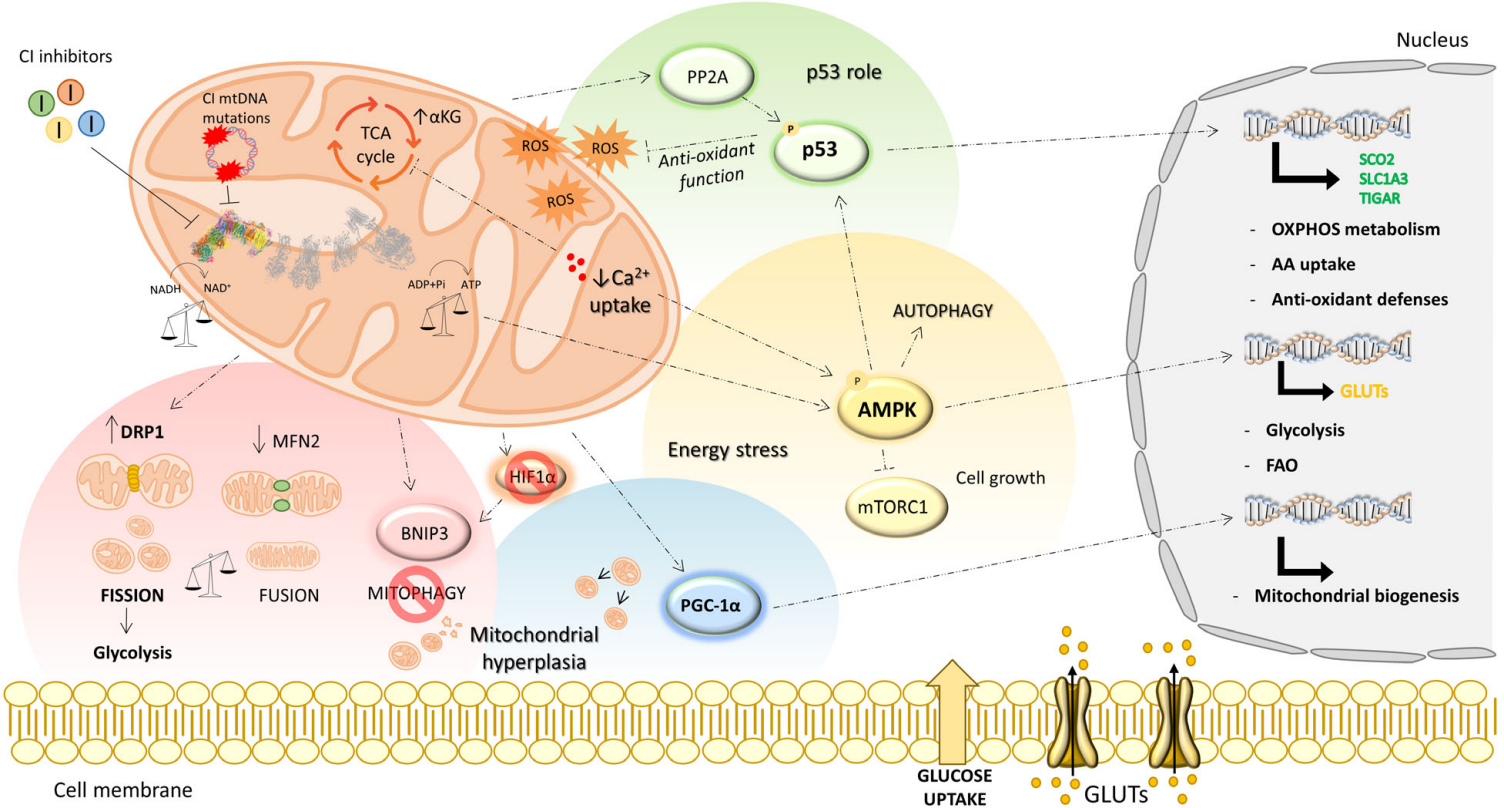
Cell‐autonomous responses triggered upon CI inhibition/ablation. Cells lacking mitochondrial CI or treated with its inhibitors develop adaptive responses to overcome the energetic and metabolic deficit. To sustain cell survival, CI dysfunction may lead to (i) an imbalance of mitochondrial dynamics toward fission, likely enhancing glycolysis; (ii) a block of mitophagy favoring the accumulation of defective mitochondria; and (iii) a PGC‐1α‐mediated compensatory biogenesis promoting mitochondrial hyperplasia. The energetic impairment also triggers mitochondria‐to‐nucleus crosstalk (retrograde signal) *via* the induction of several signaling cascades, probably favoring CI‐impaired cancer cell survival. AMP/ATP ratio increase sustains AMPK activation, thus promoting the expression of glucose transporters (GLUTs) with the subsequent increase in glucose uptake and fatty acid β‐oxidation (FAO). Further, the AMPK‐mediated inhibition of mTORC1 signaling may explain the initial block of growth observed in CI‐defective cancers. Another retrograde signaling may be induced by the alteration of mitochondrial Ca^2+^ uptake, impacting on TCA cycle rate and AMPK‐mediated autophagy process to avoid cell death. In addition, upon CI inhibition, the stimulation of AMPK and PP2A likely induces p53 activation, which may play a pivotal role in metabolic adaptation. Indeed, p53 allows to overcome the energy dysfunction supporting the mitochondrial respiration through the expression of SCO2, a regulator of complex IV assembly. The activation of p53 may also help to relieve amino acid supply by transactivating the carrier SLC1A3 expression. Finally, the positive regulation of TIGAR from p53 confers to CI‐defective cancer cells a cytoprotective effect safeguarding them against ROS overproduction.

A functional CI is crucial for the generation of mitochondrial membrane potential, the consequent ATP production, and the maintenance of cellular redox state [22, 23]. CI dysfunction is followed by a series of ancillary side effects including energy deficiency, redox equilibrium disturbance, and metabolic remodeling [24, 25]. Indeed, it has been demonstrated that cells lacking CI or treated with metformin experience a profound metabolic reprogramming including increased glucose consumption and lactate production, suggestive of an upregulated glycolysis [11, 26, 27, 28]. Moreover, the TCA cycle is predominantly replenished through anaplerotic reactions fed by glutaminolysis and TCA cycle intermediate balance is altered [11, 26, 27, 28]. In this context, the accumulation of α‐ketoglutarate (αKG) and of intramitochondrial oxygen favor the degradation of hypoxia‐inducible factor 1α (HIF‐1α), the key regulator of hypoxic responses, thus reducing cancer growth both *in vitro* and *in vivo* [6, 11, 20, 21]. Under the pressure of adverse microenvironmental conditions, such as nutrients and oxygen restriction, compensatory cell‐autonomous responses could be triggered by cancer cells to ensure survival and proliferation, counteracting these side effects (Fig. 1) [29]. For example, besides its role in the regulation of HIF‐1α stability, the pleiotropic metabolite αKG is known to regulate several enzymes, including those involved in histone and DNA demethylation [30]. In this frame, high levels of αKG found in CI‐defective models may modify the epigenomic setting of cancer cells, thus representing a metabolic signal for the activation of adaptive responses. The molecular mechanisms underlying cell‐autonomous adaptive responses have not been fully clarified and might be particularly relevant during cancer progression when cancer cells are exposed to hypoxic and metabolic constraints (Fig. 1).

### Mitochondrial dynamics, mitophagy, and biogenesis as checkpoints of CI‐defective cancer fate

To prevent cell damage and death responses and to compensate the energetic deficit that follows CI dysfunction, mitochondrial quality control is often activated [31]. Strongly depolarized and damaged mitochondria are subjected to a selection in which a balance between dynamic processes (fission and fusion), organelle elimination (mitophagy), and replacement (biogenesis) must be maintained [32, 33] (Fig. 1). Some studies report an imbalance of mitochondrial dynamics toward fission in CI‐mutated cells and tumors [34, 35]. In particular, increased levels of dynamin‐related protein 1 (DRP‐1) and mitochondrial hyperplasia were observed in oncocytic thyroid and in type I endometrial carcinomas harboring high loads of disruptive CI mutations [34, 35]. Moreover, metformin‐mediated CI inhibition was shown to cause a decrease in the expression of the fusion regulator mitofusin 2 [36], supporting the hypothesis that when CI dysfunction occurs, mitochondrial dynamic processes are skewed toward fission, resulting in a massive mitochondrial fragmentation and a likely enhancement of glucose catabolism that may sustain cell survival [37, 38]. Fragmented mitochondria with reduced membrane potential are recognized by mitochondrial quality control systems and eliminated by active mitophagy [39]. Indeed, in type I endometrial carcinoma an upregulation of the mitophagy regulator BCL2‐interacting protein 3 (BNIP3) was observed [40]. In this frame, it has been recently shown that CI activity is essential for a full mitophagy activation upon pharmacologic and genetic inhibition [41]. Moreover, renal oncocytomas bearing mutations in *MT‐ND4* and *MT‐ND5* showed a defective autophagy program in which mitophagy players are primed, but the final steps of this process are not executed [42]. It is also known that HIF‐1α triggers mitophagy as an adaptive response to hypoxia through BNIP3 activation, suggesting that the prevention of mitophagy and the triggering of mitochondrial hyperplasia are favored in CI‐defective cancer models where HIF‐1α destabilization occurs [43]. A peculiar case is represented by metastatic oncocytic thyroid XTC.UC1 cells in which the co‐occurrence of m.3571insC/*MT‐ND1* and Parkin defects determines the accumulation of aberrant mitochondria because of an inefficient mitophagy program [44]. In this context, mitochondrial proteases play a major role in executing mitophagy. These enzymes are also essential in warranting mitochondrial plasticity and fitness, while some of them provide a direct control of respiratory complexes, including CI, by regulating the import and the turnover of several structural subunits [45]. In particular, the *m*‐ and *i*‐AAA proteases are responsible for the degradation of membrane‐embedded proteins [46, 47], while LONP1 and caseinolytic protease P (ClpP) promote the proteolysis of the peripheral arm of CI, hence limiting ROS production in depolarized mitochondria [48, 49]. LONP1 has been also found upregulated in several solid cancers where it seems to promote aggressiveness through increased ROS production via CI [50, 51, 52, 53], while its depletion hampered tumor growth [50, 52, 54]. Interestingly, both upregulation and repression of LONP1 induced a metabolic rewiring and a glycolytic activation in murine melanoma cells. However, its overexpression has been proposed to induce a metabolic reprogramming that involves both anabolism and catabolism through the regulation of N‐ and Q‐modules of CI, ultimately leading to cell survival. In an opposite fashion, the downregulation of this protease affects CI assembly, prompting a generalized mitochondrial dysfunction and a metabolic switch toward catabolism that induces cell senescence [50]. In this context, pharmacological targeting of LONP1 may represent an intriguing and unexplored way to indirectly impact on CI stability and consequently cancer cell metabolism, also considering that the protease inhibition has been found effective to selectively induce apoptosis in cancer cells [55]. Conversely, hyperactivation of ClpP in cancer cells degrades subunits of respiratory chain complexes, predominantly of CI, inducing a bioenergetic defect and suppressing tumor progression [56]. Interestingly, ClpP has been found overexpressed in type I endometrial carcinoma carrying pathogenic mtDNA mutations and CI dysfunction, thus supporting the possibility of an enhanced CI degradation in these models [34]. In the attempt to rescue the reduced energetic competence of CI‐defective cells, a compensatory biogenesis can be stimulated through the activation of proteins belonging to the peroxisome proliferator‐activated receptor gamma coactivator 1 (PGC‐1) family [57]. The most striking example is represented again by oncocytic tumors where the activation of pro‐biogenesis signals is thought to cause a compensatory but short‐circuited massive accumulation of aberrant mitochondria by the transcription complex containing PGC‐1 related coactivator (PRC) and estrogen‐related receptor α (ERRα) [58, 59, 60]. This phenotype, together with a PGC‐1α upregulation, is also observed both *in vivo* and *in vitro* in genetically CI‐defective cancer models and in CI‐mutated type I endometrial carcinomas [11, 40, 61, 62]. However, it has been also reported that cancer cells harboring a nonsense mtDNA mutation in *MT‐ND5* displayed a likely ROS‐mediated compensatory mitochondrial biogenesis orchestrated by PGC‐1α upregulation, which in turn favors cancer cell growth [63]. Similar to other players regulating cancer progression, PGC‐1α expression levels seem to be associated with distinct cancer stages [64]. Nevertheless, the overexpression of PGC‐1 protein family members is linked with the peculiar benign behavior of oncocytomas, although in other contexts it has been found to foster invasiveness. Indeed, Andrzejewski and colleagues showed that PGC‐1α can promote invasive features enhancing the bioenergetic capacity of breast cancer cells *in vivo*, facilitating their ability to cope with biguanides such as metformin. In this context, the inhibition of CI by metformin did not affect the metastatic potential of cancer cells since high levels of PGC‐1α support it by promoting glycolytic metabolism and diverting mitochondrial metabolites usually employed for OXPHOS‐dependent ATP production toward anabolic reactions [65]. Moreover, the upregulation of mitochondrial biogenesis mediated by PGC‐1β has been found to completely rescue the OXPHOS defects caused by a homoplasmic missense CI mutations in *MT‐ND2* in *cis*platin‐resistant lung cancer cells [66]. These findings highlight the ability of cancer cells with severe CI mutations to overcome respiration impairment by activating biogenesis as an adaptive response to support cell survival [66].

Overall, the equilibrium between mitochondrial dynamics, mitophagy, and biogenesis is involved in different phases of tumor progression as secondary effects of CI impairment. In a first phase, they may contribute to the arrest of cancer growth observed in CI‐defective tumors, but lately they may trigger adaptive responses to overcome the metabolic stress and thus support cancer cell survival and proliferation.

### Mitonuclear signaling as compensatory pathway under CI‐mediated metabolic deficit

A tight and orchestrated communication between nDNA and mtDNA genomes is required to adapt mitochondrial function in the presence of the continuous changes that occur in both intracellular and extracellular microenvironments. Multiple pathways coordinate such mitonuclear communication during both physiological and pathological processes [67]. This crosstalk can be driven by the nucleus (anterograde signal) with the production of nuclear‐encoded mitochondrial proteins that regulate mtDNA gene expression resulting in a maintenance of mitochondrial activity. However, upon different stressors, mitochondria can produce signals (retrograde signal) that can impact on both cytosolic and nuclear targets modifying gene expression profile as well as protein abundance and localization [67]. In CI‐defective cancer cells, the concomitant energetic impairment and the failure of quality control mechanisms are found to trigger a compensatory response activating the retrograde crosstalk [68], whose main signals are mediated by the AMP/ATP ratio, altered Ca^2+^ homeostasis, and ROS production [67] (Fig. 1).

#### Energetic stress

Severe CI mutations induce a profound energetic crisis followed by the activation of the main energy sensor AMP‐activated protein kinase (AMPK), triggering a signaling cascade that switches on catabolic pathways while slowing down anabolism [6, 69, 70]. Indeed, upon pharmacological inhibition of CI, the activation of AMPK promotes the uptake of glucose, the expression of its transporters (GLUTs), the activation of hexokinase II, and 6‐phosphofructo‐2‐kinase to overcome ATP depletion [71, 72]. Upregulation of AMPK and stimulation of fatty acid β‐oxidation (FAO) were observed upon inhibition of CI with metformin in a preclinical models of ER^+^ breast cancer in correlation with cell dormancy and survival [73]. Overall, these data highlight the role of AMPK in promoting metabolic plasticity and adaptive responses upon CI impairment. On the other hand, AMPK is reported as negative regulator of cell proliferation and protein translation underlying an anticancer role for this kinase. A plethora of studies reported that CI inhibition by metformin triggers the AMPK signaling cascade that in turn inhibits the mammalian target of rapamycin complex 1 (mTORC1)‐mediated protein synthesis and cell growth [74, 75, 76, 77]. Despite this, reduced CI‐dependent NADH consumption caused by NDUFV1 knockdown enhanced aggressiveness in breast cancer models, resulting in an activation of protein kinase B (AKT) and mTORC1, which in turn hampered the autophagy machinery. This cascade is turned off when the NADH/NAD^+^ ratio is restored, preventing cancer invasiveness [78]. It is interesting to note that in this model a partial reduction of cellular respiration is observed, hence according to the *oncojanus* paradigm for CI dysfunction, it is plausible that such a mild defect may promote tumor progression via AMPK/AKT/mTORC1, whereas severe dysfunction leads to quiescence until other adaptative responses set in [79].

#### Ca^2+^‐dependent responses

Ca^2+^ signaling has been reported as a pivotal process that coordinates different extracellular stimuli, triggering a vast repertoire of intra‐ and inter‐cellular processes [80]. Functional mitochondria contribute to Ca^2+^ homeostasis acting as buffer organelles during cytosolic Ca^2+^ increase, and its uptake into the mitochondria is crucial to regulate ATP production. Indeed, this cation is able to modulate TCA cycle key enzymes and several components of OXPHOS, maintaining the balance between cell survival and death and activating retrograde signal responses [81, 82]. In cancer cell models, CI inhibition mediated by δ‐tocotrienol triggers mitochondrial Ca^2+^ overload and ROS overproduction, leading to the activation of mitogen‐activated protein kinase family members, such as c‐Jun N‐terminal protein kinase 1 (JNK1) that in turn can activate cell death in the form of paraptosis [83]. Similarly, treatment with biguanides also leads to a mitochondrial Ca^2+^ overload, inducing swelling and damaging organelles. Indeed, metformin treatment inhibits the opening of the mitochondrial permeability transition pore (mPTP) allowing cancer cells to overcome apoptosis [36]. This study confirmed that the anticancer effect of metformin is mainly due to inhibition of cell cycle rather than stimulation of apoptosis [36, 84, 85]. In contrast, cells carrying homoplasmic mutations in *MT‐ND5* present with reduced mitochondrial Ca^2+^ uptake [86, 87]. In particular, the m.13565C > T/*MT‐ND5* mutation in osteosarcoma 143B cybrids caused a partial mitochondrial depolarization and loss of TCA cycle enzyme regulation, thus affecting metabolism [86], while the m.13514A > G/*MT‐ND5* mutation has been associated with increased AMPK‐mediated autophagy and faster mitochondrial turnover, as a compensatory mechanism relieving organelle dysfunction [87]. Moreover, the knockdown of CI assembly factor NDUFAF3 in a breast cancer cell model provoked a decrease in both mitochondrial Ca^2+^ influx and efflux without affecting mitochondrial membrane potential [88]. This condition did not cause changes in cell proliferation but prevented cell death [88]. Altogether, these findings suggest that Ca^2+^‐mediated retrograde pathway induced by CI impairment may facilitate the survival and subsequent rescue of cancer cell proliferation.

#### ROS production

Respiratory complexes are the major intracellular source of ROS, molecules that regulate different signaling involved in both cell survival and apoptosis [89]. When the respiratory chain is inhibited, escaping electrons may generate superoxide anion and consequentially stimulate the intracellular antioxidant response [90]. Chronic ROS exposure due to stable OXPHOS dysfunction inactivates the iron–sulfur clusters of CI, complex II and complex III further increasing the impairment of mitochondrial respiration and energy production [90]. In this context, it is extremely important to correlate the degree of CI dysfunction with ROS production and their impact on tumor progression or aggressiveness. Indeed, severe homoplasmic mtDNA mutations prevent ROS generation, thus hampering tumor growth [5, 6, 7]. On the other hand, missense CI mutations mostly induce an increase in ROS generation promoting growth advantage and tumor aggressiveness through the AKT/HIF‐1α axis [7, 8, 10, 91, 92]. Intriguingly, Ishikawa and colleagues showed that cells with a low metastatic potential with wild‐type mtDNA grew significantly faster than their isogenic counterpart carrying the homoplasmic frameshift m.13885insC/*MT‐ND6*, thus confirming that mutations severely affecting CI hamper tumor growth. However, the transfer of such mutated mtDNA is associated with ROS production and increased the metastatic potential of these cells, highlighting the lack of a direct correlation between the growth rate of the primary tumor and its aggressiveness [8]. These data support that the different degrees of CI mutation severity modulate ROS production, thus differently impacting on cancer cell survival and proliferation.

### Linking p53 to metabolic adaptation in CI‐defective cancer models

Upon CI deficiency and nutrient shortage, the tumor suppressor gene *TP53* may play a crucial role in contributing to metabolic adaptation, as it modulates the balance between oxidative and glycolytic pathways [93]. Indeed, p53 has been found to positively regulate the complex IV assembly factor synthesis of cytochrome *c* oxidase 2 (SCO2), which underlies a role for p53 in enhancing mitochondrial respiration [94]. The stress‐sensing capability of p53 was originally described in the context of genotoxic stress through phosphorylation on serine 15 by DNA‐dependent protein kinase (DNA‐PK) and ataxia telangiectasia‐mutated (ATM) kinase, which disrupts the p53‐MDM2 interaction and enhances its activity and stability. Serine 15 phosphorylation is also provided by the activation of AMPK that follows CI damage or glucose deprivation, suggesting that the same p53 targets induced under genotoxic stress could be induced in CI‐deficient cancer cells, causing a persistent cell proliferation block [70, 95]. Interestingly, nutrient shortage has been shown to induce p53 upon the activation of the protein phosphatase 2 (PP2A)‐B55α complex [96]. It is known that metformin‐mediated CI inhibition triggers the activation of PP2A, leading to a metabolic collapse and apoptosis [97]. Although in the latter study, p53 was not called into play, it would be interesting to understand whether it may have a role in deciding the cell fate during a severe metabolic crisis. In particular, active glycogen synthase kinase 3β (GSK3β) has been shown to be a key determinant of the energy collapse, and this protein may act both as a negative and a positive p53 regulator [98, 99]. P53‐null or some mutant cancers may be more sensitive to the combo of metformin and hypoglycemia than those with a wild‐type p53 since the latter may be activated by both AMPK and GSK3β and attempt a metabolic compensation by fostering OXPHOS. Besides the effect on cellular bioenergetics, CI is required for aspartate synthesis, so that CI deficiency causes auxotrophy for an amino acid that is necessary for nucleotides and protein synthesis [100]. In this context, the possible activation of p53 that follows a metabolic crisis due to CI impairment may come to help to relieve amino acid shortage, for example, by transactivating the glutamate/aspartate carrier SLC1A3 expression [101]. P53 may also contribute to cell responses to oxidative stress, which is established in cells carrying a mild CI dysfunction. Promoters of p53‐regulated genes with antioxidant functions appear to be sensitive to low levels of p53, whereas pro‐oxidant p53 target genes are activated in response to higher p53 levels upon more extensive stress [102]. In this context, p53 has been also shown to regulate TP53‐induced glycolysis and apoptosis regulator (TIGAR) and apoptosis‐inducing factor (AIF), protecting cancer cells against ROS‐induced death [103, 104]. Overall, a role for p53 in CI‐defective neoplasms warrants investigation, as it may reveal crucial in the adaptive responses that allow survival or persistence of cancer cells during a metabolic crisis, such as in the case of indolent oncocytomas with a disassembled CI, where this tumor suppressor does not appear to be ever the main driver of tumorigenesis (Fig. 1).

## Tumor microenvironment‐related adaptive responses

**Fig. 2:**
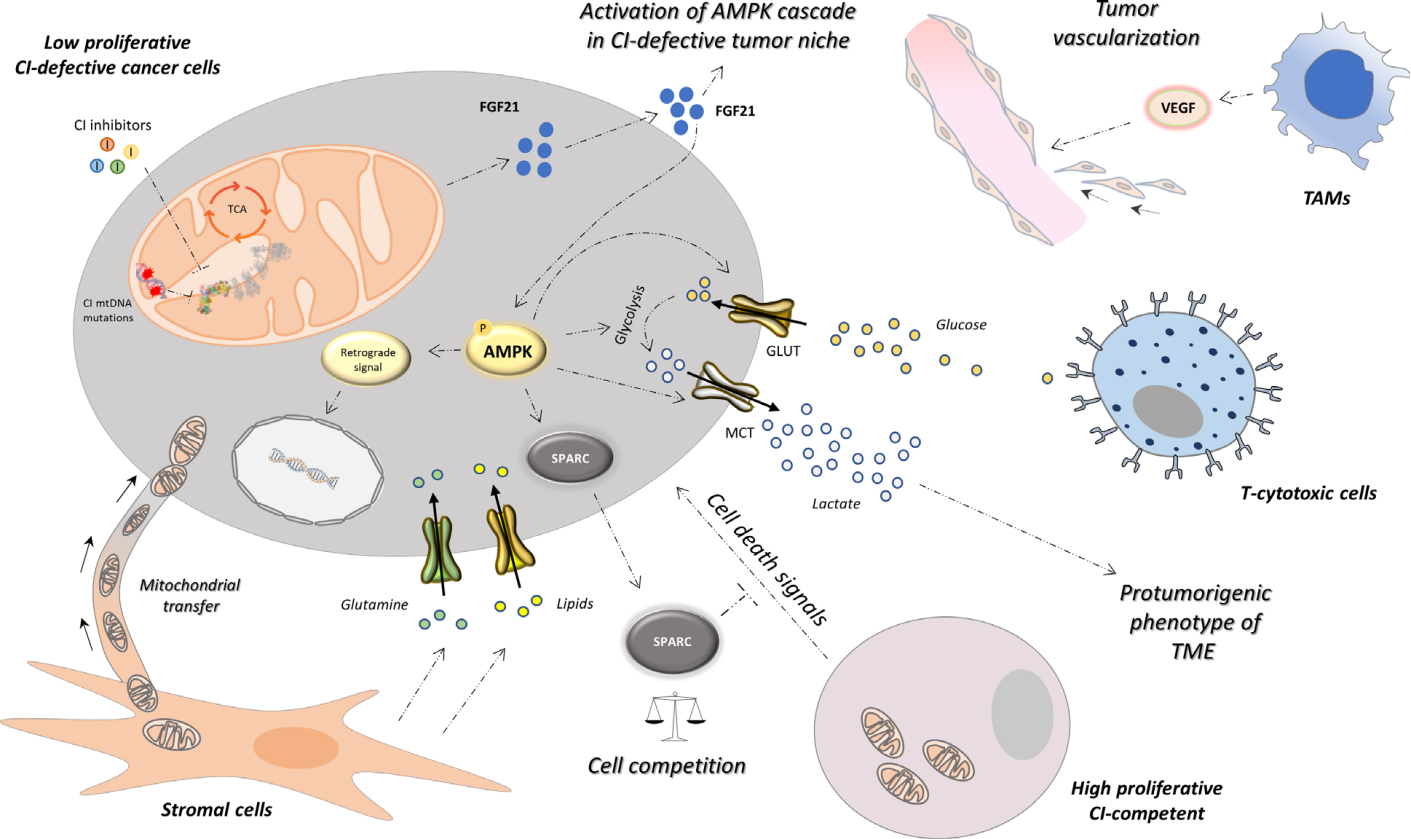
Non‐cell‐autonomous responses triggered upon CI inhibition/ablation. Following an initial block of proliferation, CI defect may activate various non‐cell‐autonomous mechanisms to overcome the bioenergetic deficit, by educating neighboring non‐malignant cells to promote cancer cell survival. In particular, the activation of AMPK triggers a series of molecular and metabolic pathways that allow CI‐defective cells to increase the uptake of nutrients (e.g., glutamine, lipids, and glucose), restricting nutrient availability in the tumor *milieu* for cytotoxic T cells and thereby reducing their defensive potential. Furthermore, the elevated glucose uptake fosters glycolysis, increasing lactate production and release, which in turn induces an acidification of the tumor niche blocking the antitumorigenic capacity of various immune cells. In CI‐defective tumors, differentiation of M2 TAMs is induced, reducing cytotoxic effects, promoting pro‐angiogenic cytokines release (i.e., VEGF), and supporting tumor neovascularization. Besides immune cells, tumors harbor resident fibroblasts, which can reverse the energetic deficit of CI‐ablated cancer cells by directly providing ‘healthy’ mitochondria through nanotubular or vesicular transfer. In addition, CI‐defective cells were shown to stimulate the release of *mitokines*, among which FGF21 may regulate energy metabolism by activating AMPK in the tumor niche and amplifying these prosurvival signals in autocrine and paracrine manners. Finally, CI‐defective cancer cells may be involved in cell competition phenomena as 'loser' cells, due to their low‐proliferative rate. In this scenario, AMPK activation may trigger SPARC secretion blocking death signals released by the neighboring CI‐competent fitter cancer cells.

Since TME is a well‐known supporter of cancer invasiveness and progression, it is not surprising that adaptive responses activated by CI dysfunction may include non‐cell‐autonomous phenomena. It is possible to hypothesize that signals derived from specific mitonuclear crosstalk reach the extracellular microenvironment and act as local paracrine mediators on neighboring cancer and stromal cells that in turn sustain the tumor mass (Fig. 2).

### Tumor‐stroma metabolic crosstalk in support of CI‐defective cancer cells

In recent years, merging the research fields of cancer metabolism and TME has resulted in a bulk of data on metabolite exchange between cancer and non‐malignant cells within a tumor mass. A wide collection of reports indicates that such crosstalk not only promotes metabolic reprogramming of cancer, but also influences its progression, stimulating migration, differentiation, survival, or self‐renewal [105]. We have recently shown that CI‐defective cancer cells may directly rely on protumorigenic immune cell functions. In particular, CI‐deficient solid cancer models were shown to trigger tumor‐associated macrophage (TAM) abundance, which correlated with increased myeloid‐derived angiogenesis [11]. Our findings suggest that such mechanism may compensate for HIF‐1α destabilization in CI‐deficient cancer model, laying the groundwork to future investigation on combinatorial strategies based on the synergistic inhibition of mitochondrial activity on one side and TME‐mediated adaptive response on the other [11]. Moreover, cancer cells were shown to uptake metabolites from neighboring cells in a symbiotic or parasite‐like manner to thrive. Thus, the proliferation of cancer cells with severe mitochondrial defects may be supported by metabolites deriving from the tumor stroma [106]. In particular, stromal components can contribute to the survival of CI‐defective cancer cells, fostering complete nutrient exploitation and favoring the oxidative metabolism of lipids and glutamine at the expense of glycolysis [107]. Moreover, since increased glycolysis in CI‐deficient cancer cells inevitably leads to acidification due to lactate secretion, it may be envisioned that targeting CI in cancer may trigger various TME protumorigenic effects known to be activated by this metabolite [108]. Finally, the high glycolytic flux in CI‐deficient cancer cells also results in a profound glucose depletion within the tumor mass, which impacts on the activity of immune cells in TME, as their cytotoxic function is largely fueled by glucose [109] (Fig. 2).

### Mitochondrial transfer as a mechanism to compensate OXPHOS deficiency

Canonically, horizontal gene transfer is well known to occur in bacteria and archaea, whereas higher organisms including mammals are mainly characterized by vertical transfer mechanisms [110, 111]. However, respiration‐deficient cancer cells seem to break these rules, as it has been shown they can educate neighboring stromal cells to become providers of healthy mitochondria. In particular, cancer cells with damaged or no mtDNA that present with severely compromised mitochondrial respiration, including CI deficiency, were shown to acquire healthy mitochondria or mtDNA molecules from TME to support tumorigenesis by restoring metabolic flexibility [112, 113]. Such intercellular mitochondrial transfer may increase OXPHOS function and support *de novo* pyrimidine biosynthesis for proliferation and DNA synthesis [114, 115, 116]. Moreover, the horizontal transfer of mtDNA through extracellular vesicles was shown to guide hormonal therapy resistance by restoration of OXPHOS and exit from dormancy in therapy‐induced breast cancer stem cells [117]. As the number of tumors in which these phenomena are identified grows, it is possible to hypothesize that mitochondrial transfer could be targeted as one of the adaptive mechanisms of CI‐deficient cancer cells (Fig. 2).

### Cell paracrine responses in the maintenance of CI‐defective cancers

To date, direct evidence for mitonuclear non‐cell‐autonomous communication in CI‐defective neoplasms is lacking. However, in the presence of OXPHOS alterations, circulating molecules known as *mitokines* can be secreted by cells in response to mitochondrial stress [118]. Mitokines, such as mitochondrial‐derived peptides (MDPs), growth/differentiation factor 15 (GDF‐15), and fibroblast growth factor 21 (FGF21), have been described as important mediators of metabolic adaptation [67, 118]. Both FGF21 and GDF‐15 are considered biomarkers for mitochondrial disorders [119, 120]. In particular, FGF21 is known to regulate energy metabolism and autophagy through the AMPK and PI3K/AKT/mTOR pathway activation to protect cells from energy stress and to maintain tissue homeostasis, suggesting its possible involvement also in CI‐defective tumors [121, 122, 123]. Moreover, it is known that AMPK can directly activate the secreted protein acidic and rich in cysteine (SPARC), a matricellular protein that supports glucose metabolism in adjacent cancer cells [124, 125]. Intriguingly, SPARC has an *oncojanus* behavior during tumorigenesis. On one hand, it appears to have an antitumorigenic role decreasing the metastatic potential of cancer cells and inhibiting cancer‐associated angiogenesis [126]. On the other hand, it may positively influence the secretion of metalloproteinases (MMPs) and the activation of epithelial‐to‐mesenchymal transition (EMT) [127]. Further, SPARC is involved in a process known as cell competition, that is, a cell–cell interaction phenomenon, originally discovered in *D*. *melanogaster* and described also in human cancers, resulting in a ‘battle’ of cells with different fitness. The fittest cells (winners) trigger cell death of the surrounding unfit cells (losers) that in turn release mitogenic factors and are eliminated from the tissue, favoring the proliferation of winner cells [128]. In this scenario, SPARC provides a transient protection of loser cells from caspase activation to recover from a transient and limited stress [129, 130]. It is interesting to note that CI‐defective cancer cells show a proliferative disadvantage, and they are likely to behave similarly to loser cells even if apoptosis has not been observed in these models [5, 11]. This suggests that a restrain in cell competition may exist and it would be interesting to understand whether *mitokines* and/or SPARC are secreted by CI‐defective cells, triggering adaptive responses to avoid cell death while promoting a rescue of proliferation (Fig. 2).

## Toward combinatorial approaches concurrently targeting CI and adaptive responses

Several studies demonstrated that targeting CI is an effective anticancer approach [11, 131, 132, 133]. The use of the hypoglycemic drug metformin has been associated with a reduced cancer risk in type 2 diabetes mellitus patients and hence proposed for cancer treatment [134]. The pleiotropic mechanism of action of metformin includes the inhibition of CI causing a reduction in ATP/ADP ratio and consequent AMPK activation [133, 135, 136]. Downstream of AMPK, mTOR signaling pathway, is shut off by metformin, reducing tumor growth and increasing antiangiogenic effects [137, 138, 139]. A recent study described an AMPK‐independent mechanism of action for metformin, which in turn regulates the transcriptional program of FAO genes, suggesting that besides OXPHOS other metabolic pathways may be affected by such treatment [140]. On the other hand, it is well known that metformin influences other mechanisms independently from the inhibition of CI, which are extensively discussed elsewhere [141]. Moreover, the antitumorigenic effects of biguanides may be also due to a CI‐unrelated action on TME [142]. These drugs have been shown to reduce the nuclear factor kappa‐light‐chain‐enhancer of activated B cells (NF‐kB)‐dependent signaling in cancer‐associated fibroblasts and to switch TAMs polarization through decreased proinflammatory cytokine production, keeping at bay protumorigenic cancer‐associated immune cells [143, 144]. Indeed, CI inhibition is achieved only at suprapharmacological concentrations of metformin, which poses the issue of whether at antidiabetic doses CI inhibition is really involved, or if it is the prolonged use that may protect from cancer occurrence [136, 145]. A second issue is whether only certain organs might benefit from metformin anticancer properties, that is, those where the drug is preferentially accumulated due to the presence of organic cation transporters (OCTs) [146]. Experiments on xenografts expressing OCTs demonstrated that metformin exerts CI‐related anticancer effects at plasma concentrations similar to those found in diabetic patients despite its administration dose [133, 147]. However, a relevant drawback in the use of metformin is the release of lactate that is eliminated through the kidneys to ensure the maintenance of its nontoxic concentrations in the plasma [148]. This calls for attention on the administration of higher doses needed to achieve CI inhibition in the context of cancer, particularly in individuals with compromised renal functions [149]. Moreover, a clinical study based on a cohort of 40 patients with primary breast cancer showed the activation of two different metabolic responses following metformin administration. In particular, tumors belonging to a subgroup of patients displayed an increased expression of genes involved in OXPHOS, glycolysis, gluconeogenesis, and amino acid metabolism. Such subgroup of tumors was indeed also able to increase their proliferation, suggesting that metformin treatment may induce a metabolic‐mediated resistance [150]. Several studies are currently focused on the search for metformin analogues or novel compounds more specific to target CI. In this context, a broad range of CI inhibitors showed antitumorigenic potential, but not all of them are eligible for clinical translation due to their cytotoxicity [151]. As previously described, CI inhibitors capable of enhancing intracellular ROS may be risky considering the fine line between ROS pro‐ and antitumorigenic effects [152]. This is the case for BAY 87‐2243, a specific CI inhibitor identified starting from a high‐throughput screening of compounds specifically tested to target HIF‐1 [132]. The compound was shown to promote a ROS increase in a model knocked down for the ROS scavenger enzyme glutathione peroxidase 4 (GPX4), resulting in severe cytotoxicity [153]. IACS‐010759 is another clinical grade CI inhibitor derived from compounds able to reduce HIF‐1 response and to inhibit proliferation of neoplasms of the nervous system and acute myeloid leukemia (AML) derived glycolysis‐defective cells, in which the inhibition of CI promptly triggered apoptosis [131]. Xenografts derived from the same cancer cells allowed to define the right doses *in vivo* and confirmed an anticancer effect of IACS‐010759, supporting its use in a phase I study in subjects with advanced cancers and lymphoma, which was recently completed (ClinicalTrials.gov ID: NCT03291938). Additionally, promising studies demonstrated synthetic lethality in OXPHOS proficient cancer cells following a combined treatment with IACS‐010759 and phosphogluconate dehydrogenase inhibitors [154]. Indeed, the latter is a key enzyme of redox homeostasis that allows cell survival through glycolysis and glutamine reductive carboxylation, whose targeting in combination with CI inhibition severely hampers the rewiring of energy metabolism. A high‐throughput drug screening allowed to identify EVP4593 and papaverine as the two most effective compounds able to target CI and selectively kill cancer cells under glucose deprivation [155, 156]. Indeed, they both inhibit the mTOR pathway albeit only under glucose starvation, and their combination with VEGF inhibitor bevacizumab is synergistic in exerting antiangiogenic effects in animal models [155]. This work highlights the effectiveness of CI targeting in combination with inhibition of the possible adaptive mechanisms activated in response to CI deficiency. In this frame, we have shown that by preventing the recruitment of protumorigenic M2 macrophages by clodronate, the residual growth of CI‐impaired xenografts is almost completely abolished, underlying the need of combined pharmacological treatments to counteract metabolic plasticity in cancer cells [11].

## Conclusions

The findings here retraced support the hypothesis that during cancer onset and progression, a severe mitochondrial CI dysfunction can result as a drawback for cell growth and survival. In the initial stage of tumorigenesis, the severe metabolic and energetic defects induced by CI impairment block tumor progression by keeping cancer in a low‐proliferative/quiescent state similarly to benign oncocytomas. In this frame, targeting CI represents a strategy to selectively hit cancer cells which must cope with nutrient and oxygen restriction and, consequently, adapt to progress toward malignancy. However, under selective pressures, the high plasticity of cancer cells endows them with the ability, over time, to bypass the control check points and to trigger adaptive responses, both cell‐autonomous and non‐cell‐autonomous (Figs 1 and 2). In this context, it can be hypothesized that these outcomes could allow a selection of OXPHOS‐deficient cancer cells capable to undertake alternative mechanisms to overcome and compensate for metabolic constraints and deal with changes in TME. Impeding the triggering of such mechanisms may result in synthetic lethality with defective CI, blocking the recolonization of the tumor mass with the fittest cells, and finally providing more efficient combinatory therapies to challenge the ever‐evolving faces of cancer.

## Conflict of interest

The authors declare no conflict of interest.

## Author contributions

LI, GG, and AMP conceptualized the idea for the manuscript. M.S., M.D.L., S.L., M.I., L.B., and IK drafted the manuscript. M.S., S. Mig., and S. Mil. drew the figures. MS, LI, GG, and AMP evaluated the manuscript and improved the content.
